# Editorial: Neuroscience and emotional design

**DOI:** 10.3389/fnhum.2025.1591488

**Published:** 2025-04-02

**Authors:** Qing-Xing Qu, Vincent G. Duffy

**Affiliations:** ^1^Department of Industrial Engineering, School of Business Administration, Northeastern University, Shenyang, China; ^2^School of Industrial Engineering, Purdue University, West Lafayette, IN, United States

**Keywords:** neuroscience, emotional design, affective computing, Kansei engineering, cognitive neuroscience

## 1 Introduction

More recently, few investigations have been done on neuroscience measurements to grasp the emotional needs from consumers, such as, electroencephalography (EEG) (Liu et al., 2024), event-related potentials (ERPs) (Guo et al., 2020), eye movements (Qu and Guo, 2019), functional near-infrared spectroscopy (fNIRS) (Guo et al., 2024), heart rate variability (HRV), and electromyography (EMG) (Caruelle et al., 2019). However, uncertainties and ambiguities still exist in verbalizing emotions by using physiological measurements. Further studies are still essential in Kansei data multimodal measurement with the collaboration of psychological questionnaires and various physiological measurements.

The aim of this Research Topic of Frontiers in Human Neuroscience is to introduce advanced neuroscience methods in emotional design research. The Research Topic embraces the application of advanced neuroscience methods in emotional design to generate new insights and shed new light on existing methodologies and theories. With this aim, we collected five original research articles focusing on different aspects in emotional design with neuroscience measurements.

Ya-Feng et al. introduced Magilock, a pre-locked mechanism designed to address the “Midas touch problem” in eye-tracking systems, where accidental activations occur due to unintended gaze. Through ergonomic experiments with 20 participants, the researchers determined that a 200 ms lock and unlock time optimizes control accuracy and user experience. By requiring users to fixate on a target and confirm with a secondary input (e.g., a keyboard press), Magilock significantly reduced accidental triggers, making it particularly useful for applications like assistive technology and gaming where precision is critical.

Ding et al. investigated how the gender of virtual chatbots influenced user attention and usage intentions. Using EEG/ERP techniques and subjective questionnaires with 31 participants, the study found that female chatbots elicited stronger neural responses (larger P100/P200 amplitudes) and higher usage intentions across genders. Female participants showed a preference for female chatbots, while male participants allocated more attention to male chatbots (larger N100). These findings suggest that chatbot designers should consider gender alignment and societal biases to enhance user acceptance and interaction quality.

Cao et al. explored the cognitive effects of fragmented reading, where users rapidly switched between short, dissimilar texts. Using EEG/ERP experiments (focusing on the P200 component) and digit-span tasks with 23 participants, the researchers found that high text dissimilarity reduced working memory capacity and increases cognitive load, while low dissimilarity (cohesive content) minimized these negative effects. The results suggest that organizing digital content cohesively can mitigate the cognitive strain caused by fragmented reading, improving users' ability to retain and process information.

Tian et al. demonstrated how miners' personality traits and emotional regulation strategies influence job burnout and risk preferences. Using surveys and eye-tracking experiments with 50 male miners in China, they found that neuroticism predicts burnout, while expressive suppression mediates and moderates relationships between personality traits and burnout. Miners with low emotional exhaustion preferred ambiguous risks, showing focused gaze patterns, whereas those with high exhaustion chose safer options. The findings highlight the role of emotional regulation in reducing burnout and improving safety decisions.

Shen et al. aimed to study how home environment features influence restorative potential and neural responses using questionnaires and Neu-VR. Key restorative characteristics include favorable window views, light warm colors, spacious rooms, and outdoor access. Environments with these features elicited positive neural responses, such as reduced pupil dilation and lower attention shifts, indicating relaxation. The findings suggest these elements enhance wellbeing and offer objective measures for evaluating restorativeness. The study contributes to evidence-based design for restorative homes but calls for further research to validate neural indicators and their broader applicability.

## 2 Conclusion

These studies integrate neurophysiological tools (EEG, eye-tracking) with behavioral experiments to address challenges in emotional design. They emphasize data-driven insights for optimizing design, usability, and safety across diverse contexts. We hope this Research Topic can act as a resource for those interested in this Research Topic, trigger further discussion, and eventually push forward development in this area.
